# Managing mental health disorders: Experiences of primary care providers in rural South Africa

**DOI:** 10.4102/phcfm.v17i1.4979

**Published:** 2025-08-26

**Authors:** Petra J. Bouwer, Louis S. Jenkins, Johann Schoevers

**Affiliations:** 1 Department of Family and Emergency Medicine, Faculty of Medicine and Health Sciences, Stellenbosch University, Cape Town, South Africa; 2 Primary Health Care Directorate, Department of Family, Community and Emergency Care, Faculty of Medicine and Health Sciences, University of Cape Town, Cape Town, South Africa; 3 Department of Family and Emergency Medicine, George Hospital, Western Cape Department of Health, George, South Africa

**Keywords:** mental health, primary care, experiences, rural district, South Africa

## Abstract

**Background:**

Mental health disorders are increasing globally. In South Africa, primary healthcare (PHC) services are tasked with mental healthcare, with limited resources. A task-sharing approach between PHC role-players has also been met with barriers, including negative attitudes towards mental health care, organisational constraints and insufficiently trained staff.

**Aim:**

To assess the perceptions and experiences of PHC practitioners in managing common mental health disorders.

**Setting:**

Primary healthcare facilities in the Garden Route District, South Africa.

**Methods:**

An observational, descriptive study using a cross-sectional survey obtained a representative sample of 130 participants. Redcap© platforms captured data, which were analysed to give frequencies and means using simple descriptive statistics.

**Results:**

Most participants (68.46%) reported average or below average competence in managing mental health conditions. Out-Patient Departments (OPDs) (68.42%) and PHC clinics (56.25%) found reaching a referral practitioner to be challenging. Waiting times of referred patients were longer at hospital OPDs and clinics than at Community Day Centres.

**Conclusion:**

Resources allocated to PHC mental health services remained inadequate, while available support structures were underutilised. The presence of a dedicated mental health practitioner at a facility had a direct influence on the experience of the staff in managing these disorders. Policy makers and managers should motivate for training in mental health and empower the PHC system to offer acceptable mental health services, in accordance with national and international guidelines.

**Contribution:**

This research contributed insights into the current mental health ecosystem in primary care, and the need for increased awareness, training and utilising available resources, which could be useful in similar contexts in sub-Saharan Africa.

## Introduction

The prevalence of mental health disorders are increasing globally and also in South Africa (SA).^1^ Primary healthcare (PHC) practitioners play an important role in identifying, diagnosing and managing patients with common mental health disorders.^2^

In 2016, it was estimated that 14.8% of the world’s population suffered from mental health and substance use disorders.^3^ The recent coronavirus disease 2019 (COVID-19) pandemic increased the prevalence of mental health disorders even further, with a global prevalence of 28.0% reported for depression alone.^1^ Although neuropsychiatric disorders are the third highest contributors to SA’s burden of diseases, there has not been sufficient allocation of resources to address these.^2,4,5,6^ Less than 3.0% of the national health budget has been directed towards mental health services in SA, which is similar in other low- and middle-income countries.^3^ It is also estimated that these conditions contribute to more than a quarter of the years lived with disability (YLD).^7,8,9^

There is an increasing demand for specialist mental health services, such as mental health nursing practitioners, psychiatrists and psychologists. Although the need to increase the specialist workforce is described in policy, recent publications reported a mere 1.6 specialists per 100 000 people in Africa.^10^ This is worrisome when compared to Europe, at 44.8 per 100 000, and the global average of 13 per 100 000. The majority of countries (79%), participating in the World Health Organization (WHO) Mental Health Atlas, allocated less than 20% of their mental health budgets to mental health services at general hospitals and even more of them (81%) allocated less than 20% to mental health in primary care facilities.^10^ In SA, these specialist practitioners include psychiatrists, social workers and psychologists, with the highest contributor being mental health nurses at 0.9 per 100 000 people.^2,10^ This places most of the service requirements on PHC practitioners. This workload shift to PHC is not supported by sufficient resources, particularly in the public health sector and especially in rural areas, resulting in overworked and insufficiently trained staff to manage both the high burden of disease and patients requiring specialist care.^2,7^ In addition, only 2% of total research output in Africa is focused on mental health.^11^ This suggests an unbalanced equation of high demand against a poorly funded and overworked PHC system.

The Western Cape province has the highest recorded lifetime and 12-month prevalence of mental health disorders (39.4%) in SA.^2,6^ This province has implemented clinical guidelines such as the Practical Approach to Care Kit (PACK), to assist PHC practitioners to manage common mental health conditions.^6^ The PACK is an integrated guideline for PHC that encourages practitioners to consider common mental health disorders in the assessment of symptoms and assist with diagnosis and management. However, various barriers exist in applying this guideline, including a high prevalence of mental health stigma, insufficiently trained staff, a high burden of disease and limited specialist support.^2,7^ The guidelines are only useful if the practitioner can identify the presence of a mental health disorder and subsequently apply the recommended management. Practitioners should be able to identify cues to a potential mental problem and be motivated to explore the possibility within a consultation.

The increasing prevalence of mental health disorders, both internationally and locally, together with the policies, plans and health improvement initiatives to alleviate this burden, should therefore be at the forefront of the health care system’s resource allocations and actions.^2,3,8,11^ If primary mental health care is part of the model of PHC, then it needs to be supported by appropriate infrastructure, medication, workforce and information. In SA, it is estimated that 75–92% of patients with mental health disorders do not receive treatment or have access to mental healthcare programmes.^2^ The workforce needs to have the capability, motivation and supportive environment required to provide this service. Person-centredness, a bio-psycho-socio-spiritual approach and continuity of care are important to recognise symptoms that indicate an underlying mental health disorder. A supportive environment implies that PHC is seen as a priority, quality is measured and improved, and specialist support is accessible and available. Primary care providers need to see improvement in the outcomes of patients who are appropriately assessed and managed.

While there are some literature that reveal challenges to the provision of primary mental health care, many issues remain unexplored, particularly in the South African context.^7^ The aim of this study was to explore some of these challenges in more depth through the perceptions and experiences of PHC providers who manage patients with mental disorders in PHC.

## Research design and methodology

### Study design

This was an observational, descriptive, cross-sectional survey.

### Setting

The setting was all 75 public PHC facilities comprised of PHC clinics, community day centres (CDCs) and district hospital out-patient departments (OPDs) within the Garden Route District (GRD) of South Africa (Box 1). These facilities are tasked with managing common mental health disorders and referring to specialist care as required.

BOX 1 Primary health care facilities per sub-district.^14^

**Table:** 

Kannaland: – Calitzdorp Clinic – Ladismith Clinic – Amalienstein Clinic – Van Wyksdorp Clinic – Ladismith Hospital – Zoar Clinic	Hessequa: – Albertinia Clinic – Slangrivier Clinic – Still Bay Clinic – Bloekombos Clinic – Riversdal Hospital OPD – Riversdal Clinic
Mossel Bay: – Mossel Bay Hospital OPD – D’Almeida Clinic – Alma Clinic – Clinic-in-Asla – George Road Clinic – Hartenbos Clinic – Eyethu Clinic – Sonskynvallei Clinic – Great Brak River Clinic – Friemersheim Clinic – Herbetsdale Clinic – Brandwacht Clinic	Oudtshoorn: – Bhongolethu Clinic – Bridgeton CDC – De Rust Clinic – Dysselsdorp Clinic – Haarlem Clinic – Oudtshoorn Civic Centre Clinic – Oudtshoorn CDC – Oudtshoorn Hospital OPD – Toekomsrus Clinic
George: – Blanco Clinic – Conville CDC – George Central Clinic – George Civic Centre Clinic – Herold Satellite Clinic – Kuyasa Clinic – Lawaaikamp Clinic – Pacaltsdorp CDC – Parkdene Clinic – Thembalethu CDC – Uniondale Clinic – Uniondale Hospital	Knysna: – Hornlee Clinic – Keurhoek Clinic – Khayelethu Clinic – Knysna Town Clinic – Knysna CDC – Knysna Hospital OPD – Sedgefield Clinic
Bitou: – Crags Clinic – Kwanokuthula CDC – New Horizon Clinic – Plettenberg Bay Clinic	

*Source:* Western Cape Government. Western Cape Clinics Directory [homepage on the Internet]. 2022 [cited 2022 Apr 13]. Available from: https://www.westerncape.gov.za/directories/facilities/944

OPD, out-patient department; CDC, community day centre.

The GRD consists of an area of 23 331 km^2^ and has an estimated population of 621 245, with an average household size of 3.5 people.^12,13^ The district has a range of rural and urban areas with 75.0% of households staying in a house on its own stand, to 9.1% staying in an informal dwelling.^12^ It is divided into the following sub-districts: Kannaland, Oudtshoorn, Hessequa, George, Mossel Bay, Bitou and Knysna.

### Study population

The study population comprised all PHC providers working at PHC clinics, CDCs and district hospital OPDs within the GRD. The OPDs were included as walk-in PHC services are offered at these facilities and doctors at these facilities offer outreach and support to peripheral PHC facilities. Participants included nurse practitioners, lay and registered counsellors, medical doctors and social workers. Medical doctors were defined as follows: Sessional and permanent medical officers (MOs), community service MOs, medical intern doctors and registrars in family medicine.

#### Inclusion criteria

Health care workers with an above-stated designation, working at the facilities within the defined district, offering the following services: Consulting, diagnosing and managing PHC complaints, including mental health complaints.

#### Exclusion criteria

Health care workers with an above-stated designation, who did not offer PHC services at the listed facilities. Mobile clinics were excluded as referral was to their local PHC facility, and this could result in duplicate data.

### Sampling and sample size

As per the GRD staffing and budget report (2020), health care workers at 53 facilities met the inclusion criteria. The total personnel were reported as 1174 within the district.^15^ When corrected for administrative staff, regional hospital staff and mobile clinic staff, it was estimated that an average of five practitioners per facility met the inclusion criteria. The study population amounted to approximately 270 individuals. To achieve a confidence interval (CI) of 95%, with a 5% margin of error, a 50% response distribution and a non-response rate of 20%, a sample size of 130 health care workers was required (see Table 1).

**TABLE 1:** Facility health care workers’ distribution and sampling.

Type of facility	Number of facilities	Estimated mean number of eligible practitioners per facility	Proportion of practitioners at this level		Number of facilities needed to sample
			*n*	%	
District hospital OPD	6	8	48	23.6	3
CDC	7	5	35	17.2	4
Clinic	40	3	120	59.1	26

OPD, out-patient department; CDC, community day centre.

### Data collection

Face validity of the questionnaire was achieved through review by an expert panel including the research supervisor, local family physician, a specialist psychiatrist at the local regional hospital and the family physician who conducted previous research on this topic. Two rounds of reviews were held between the validating team. The following adjustments were made:

- Terminology changes were made to refer to mental health disorders, rather than conditions.
- Questions assessing confidence levels regarding identification, diagnosing and managing mental health disorders were originally grouped into one question, but this was changed to separate questions to provide separate data sets that could be evaluated.
- Recommendations for changes to the title for the possibility of future publications.
- General improvements to grammar and punctuation.

The questionnaire was piloted at Mossel Bay District Hospital OPD and D’Almeida PHC Clinic, with seven health care workers, including two clinical nurse practitioners (CNPs), three MOs, one family medicine registrar and one family physician. This represented both PHC clinics and district hospital OPDs to achieve a relative representation of the study population. The piloting process was successful, with both the email link and collection device methods tested, and no further adjustments were needed to the questionnaire. As there were no adjustments required after the piloting of the questionnaire, this data were deemed viable and included in the final dataset. The managers of the selected facilities were contacted via email and telephonically, and a suitable date for data collection was arranged.

Voluntary informed consent was obtained from participants willing to partake in the study. Data collection was done through a survey-based platform using the REDCap^®^ collection system. The REDCap^®^ platform can be used in both online and offline applications. The survey was also provided to clinic and hospital managers via an email link and distributed to eligible participants. Grading questions incorporated a Likert-scale format. The survey included the following variables (see Appendix 1):

- Participant demographics limited to age, gender, designation and the facility at which employed.
- Undergraduate training in mental health care.
- Post-graduate training in mental health care.
- Continuing professional development or short courses in mental health care.
- Motivation and confidence in recognising, assessing and managing mental health conditions.
- Quantifying common beliefs regarding a mental health care user.
- Quantifying beliefs on mental health user’s impact on daily workload.
- Grading the participant’s perceptions of mental health disorder incidence.
- Participant grading of their institutional and referral resource allocation to mental health services (counsellors, staff training, outreaches and primary carer experience).
- Quantifying participant beliefs regarding their local, primary level care team to diagnose, manage and refer mental health patients correctly.
- Participant grading of available resources to assist themselves in diagnosing, managing and referring patients with mental health disorders, for example PACK.

Data collection consisted of two platforms, namely:

- Online survey completion on the REDCap^®^ data collection system via an email-based link. This link was sent to facility managers for distribution to their staff meeting the inclusion criteria. There were very few responders via the email link system, despite three reminders being sent to managers and participants at the facility.
- Offline REDCap^®^ survey completion was done through an application-based system, namely MyCap^®^. This is the official application for the REDCap^®^ data collection system.

Arrangements were made with facility managers to do on-site data capturing via the application-based system. Facilities were visited by the primary researcher over a period of three-months. The survey was offered in English, and the primary researcher was available to individual participants who gave voluntary informed consent, during working hours in their facilities, to answer any questions during the completion of the questionnaire. A non-monetary token of gratitude (food) was provided to the participants after completion of the questionnaire. If health care workers in a sub-district or facility declined participation, attempts were made to recruit participants from other facilities that complied with the inclusion criteria.

### Data analysis

The data were reviewed with the Biostatistical Department of the University of Stellenbosch. The REDCap^®^ data were exported to a Microsoft Excel^©^ document with numerical representation of the data. The data were processed and audited to exclude incomplete data sets and a data analysis document was compiled. IBM Statistical Platform for the Social Sciences (SPSS) Statistics for Windows, version 28 (IBM Corp., Armonk, New York, USA) was used for statistical analysis. Frequencies and percentages were calculated for categorical data and means and medians were calculated for continuous data.

### Ethical considerations

Ethical approval for the study was obtained from the Health Research Ethics Committee (HREC) of the University of Stellenbosch Faculty of Health Sciences (Ref no. S23/01/019). Approval was also obtained from the Western Cape provincial Department of Health and the Garden Route district office (Ref no WC_202305_011)

## Results

Most of the participants (61.54%) were working at PHC clinics, while 23.85% and 14.62% were working at CDCs and hospital OPDs, respectively (see Table 2). One sub-district had no respondents, while one sub-district accounted for 43.85% of the sample, and two other sub-districts each contributed 13 (10.0%) participants respectively. Most participants were female (83.85%), while the average age of participants was 43 years (standard deviation [s.d.] = 11). Clinical nurse practitioners (47.24%) and MOs (32.28%) were the main contributors to the study.

**TABLE 2:** Demographics of participants (*N* = 130).

Variables	*n*	%
**Gender**		
Female	109	83.85
Male	21	16.15
**Age**	Mean: 43 (s.d.: 11)	n/a
**Sub-district**		
George	57	43.85
Mosselbay	33	25.38
Knysna	13	10.00
Oudtshoorn	13	10.00
Hessequa	10	7.69
Bitou	4	3.08
**Facility type**		
Primary health clinic or Satellite clinic	80	61.54
Community day centre	31	23.85
Hospital out-patient department	19	14.62
**Designation**		
Clinical nursing practitioner	60	47.24
Medical officer	41	32.28
Professional nurse	10	7.87
Sessional doctor	5	3.94
Lay counsellor	4	3.15
Psychiatric nursing practitioner	3	2.36
Registrar	2	1.57
Registered counsellor	1	0.79
Social worker	1	0.79

s.d., standard deviation; N/A, not applicable.

Although 88.46% of participants reported that they had received undergraduate training in mental health, most participants (68.46%) reported average or below average competence in managing mental health conditions after this level of training (*p* = 0.38) (see Table 3).

**TABLE 3:** Training received by the participants.

Undergraduate training received in mental health	*n*	%
Yes	115	88.46
No	14	10.77
Unsure	1	0.77
**Type of training received**		**(*N* = 115)**
Rotation in dedicated psychiatric ward or facility.	41	35.65
Clinical rotation in psychiatry.	33	28.70
Theory and/or formal lectures (module).	20	17.39
Informal lectures or group discussions.	12	10.43
Rotation in general wards or primary care facilities.	9	7.83
**Post Graduate training received in mental health**		
Yes	36	27.69
No	94	72.31
**Type of Post Graduate training received**		**(*N* = 36)**
Module within another course or speciality	10	29.41
CPD activity or in-service training	8	23.53
Mental health course	7	20.59
Diploma in Mental Health	6	17.65
Training seminar or short course	2	5.88
Degree in Mental Health	1	2.94

CPD, Continuing Professional Development.

### Patient workload

Mental health disorders contributed significantly to daily patient loads. There was a mean daily patient load of 28.28 (s.d.:13), with an average of 5.47 (s.d.: 5.95) patients with mental health related disorders (average of one in five patients). Regarding patient workload, 46.92% of participants stated that their workload was too much to offer effective mental health screening, and 32.31% reported avoiding mental health-related complaints during a consultation.

### Staff attitudes to patients with mental health problems

More than half of the participants reported neutral to negative opinions regarding mental health awareness at their facilities. Most of these participants gave environmental factors (e.g. workload, mental health prevalence) as their reasons for this (72.50%, *p*: 0.018). Forty-five per cent of participants reported positive emotions when presented with a patient with a mental health complaint or disorder, with most stating personal reasons (50.85%) as motivation for their emotion (e.g. own mental health status or opinion, confidence level).

### Training in mental health

Undergraduate training in mental health disorders was reported to be insufficient, with an overwhelming majority of participants (91.54%) reporting that they would attend training opportunities with regards to mental health disorders. Most participants (72.31%) did not receive any postgraduate training in managing patients with mental health disorders. Mental health guideline usage was poor. Most participants reported an average confidence level in recognising, diagnosing and managing patients with mental health disorders. Although the level of confidence reported among participants was similar in these three aspects of care, 22.45% (*p* < 0.01) reported confidence in managing patients with mental health disorders but reported low confidence in the ability to diagnose these disorders. Most participants (91.54%) were aware of the PACK-guidelines. In participants who reported low confidence in diagnosing mental health disorders, 71.42% consulted the PACK-guidelines only sometimes or never. There was a higher prevalence of regular PACK-guideline usage among participants with a high level of confidence in managing mental health disorders (46.38%) compared to participants reporting low levels of confidence (28.58%, *p*: 0.06).

The presence of a mental practitioner at a facility had a direct influence on the overall management and referral of patients with mental health disorders. In general, participants reported good experiences in referring patients with mental health disorders to the next level of care, with two sub-districts recording the highest percentages of positive experiences, while one sub-district reported the highest ‘poor experience’ (30.7%, *p*: 0.07) in relation to the other sub-districts. Participants in hospital OPDs (68.42%) and PHC clinics (56.25%) found reaching a referral practitioner to be challenging, while participants in CDCs (54.84%) found it easy for the patient to reach the next level of care or practitioner (*p*: 0.12).

## Discussion

This study explored the perceptions and experiences of PHC practitioners in managing common mental health disorders in the GRD. The key findings included: Mental health disorders contributed significantly to daily patient loads, without the required resources allocated to PHC services; undergraduate training in mental health disorders was insufficient; negative perceptions about mental health screening and interventions were barriers to the integration of mental health services in PHC; there was a positive emotion regarding mental health disorders when associated with personal experience with a mental health disorder and a positive referral experience by most; and PACK-guideline usage in managing patients with mental health conditions was poor.

A one-in-five average prevalence of mental health disorders on daily patient loads was reported in this study. This was a significant amount, requiring a knowledgeable, experienced, motivated and supported health care system. European countries have responded to this, but African countries, and in particular the Western Cape province who has the highest prevalence of mental health disorders in SA,^2,6^ have not managed to achieve close to global averages of mental health specialist distribution in their populations. In SA, it is reported that only 7.9% of the mental healthcare budget is allocated to PHC services with a public service psychiatrist distribution of 0.31 per 100 000 population.^2^ This is despite previous service package requirements that recommended 1.03 psychiatrists and 10.36 nurses providing mental health services per 100 000 population. The sub-district in this study, with a population of 140 075, had one mental health nursing practitioner, who at the time of this study was only able to service the hospital and booked patients at the OPD.

Although there have been investigations into the sharing of psychological interventions and responsibilities in PHC in SA to mitigate the shortfall of specialist mental health practitioners, this has been met with various barriers.^2^ One of these barriers was the low number of trained staff. A concerning finding in this study was that current undergraduate programmes did not seem to have prepared study participants sufficiently to identify and manage mental health disorders effectively. It therefore appears that a mismatch is occurring between the mental health service needs and adequately trained and staffed human resources.^2^

Negative perceptions and beliefs about mental health screening and interventions translated directly into suboptimal patient care, evidenced by the fact that a third of participants admitted to avoiding mental health complaints within a consultation and that almost half of the health care workers reported neutral opinions or insufficient mental health awareness at their facilities. However, a different picture was presented at the CDCs, who had dedicated and regular specialist mental practitioners on site. There was a positive approach to mental health disorders when the participants experienced support and ease of referring to a dedicated service provider for mental health conditions within their facility. The direct impact that these practitioners have on the morale of the staff and the overall experience of managing patients with mental health disorders cannot be ignored.

Despite the availability of the PACK guidelines, the low confidence levels among participants did not translate into increased usage of these guidelines as support. In fact, the participants who reported higher confidence levels in managing these conditions were consulting the PACK-guidelines the most, which was encouraging. As environmental factors such as excessive workload were the main reasons provided by participants expressing negative emotions towards managing patients with mental healthcare conditions, it is postulated that this might also had been a contributing factor to the lower rates of guideline usage.^17^ Despite previous research recommendations to strengthen guideline utilisation, this did not appear to be the case in this study.^2,18^

A training needs assessment, conducted in the Cape Metropole in 2022, reported higher training needs in child mental health (30.4%) and mental health (19.3%) as compared to other training domains such as child health or emergency care.^16^ The participants in our study supported this finding, with 91.54% reporting that they would attend training in mental health disorders.

### Strengths and limitations

A strength of this study was that a representative sample of the PHC system was obtained, namely clinics, CDCs and district hospital OPDs, to understand PHC workers’ experiences with managing patients with mental health disorders in PHC.

Although the required sample size was reached, there was not adequate representation from one sub-district. While the study was done in only one district, which limits the generalisability of the findings, there may be useful applications for similar contexts in sub-Saharan Africa.

Qualitative data would have given deeper insight into why participants held certain views or responded the way they did.

### Recommendations

Undergraduate programmes should strengthen their focus on mental health disorders, including increased practical exposure to prepare future PHC staff for the increasing burden of disease. Staff require additional training in mental health disorders, and managers need to emphasise and monitor the usage of guidelines provided to assist in managing mental health disorders. Mental health awareness and continued destigmatisation needs to be at the forefront of PHC employees. Mental health nursing practitioners play an invaluable role in the PHC system. There is a need to increase the number of these practitioners, especially to support PHC clinics.

## Conclusion

Although mental health disorders contribute significantly to daily patient loads and showing increasing trends globally, allocated resources remain inadequate. Participants tasked to integrate mental health services in PHC reported inadequate training and low confidence in managing mental health disorders, while specialist practitioners were rare and limited by PHC patient workloads and ease of referral. Resources such as the PACK guidelines were not being fully utilised. The presence of a dedicated mental health practitioner at a facility correlated positively with the experience of the staff in managing these disorders. Policy makers and managers should motivate for training in mental health and empower the PHC system to offer acceptable mental health services, in accordance with national and international guidelines.

## Appendix A - Questionnaire

### Managing mental health disorders in primary healthcare: a cross-sectional survey of the perceptions and experiences of primary care providers in the Garden Route District of South Africa

Dear participant,

We would like to invite you to take part in a research project. Please take some time to read the information presented here, which will explain the details of this project. Please ask the study staff or doctor any questions about any part of this project that you do not fully understand. It is very important that you are completely satisfied that you clearly understand what this research entails and how you could be involved. Also, your participation is entirely voluntary, and you are free to decline to participate. In other words, you may choose to take part, or you may choose not to take part. Nothing bad will come of it if you say no: it will not affect you negatively in any way whatsoever.

Refusal to participate will involve no penalty or loss of benefits or reduction in the level of care to which you are otherwise entitled. You are also free to withdraw from the study at any point, even if you do agree to take part initially.

The Health Research Ethics Committee at Stellenbosch University has approved this study. The study will be conducted according to the ethical guidelines and principles of the international Declaration of Helsinki, the South African Guidelines for Good Clinical Practice (2006), the Medical Research Council (MRC) Ethical Guidelines for Research (2002), and the Department of Health Ethics in Health Research: Principles, Processes and Studies (2015).

What is this research study all about?This study aims to evaluate the the perceptions and experiences of primary care providers to manage common mental health disorders.

Why do we invite you to participate?You were identified as a primary care provider offering services within the Garden Route District.

The survey will take approximately 20minutes to complete. By clicking next you are consenting for this data to be used in the study as indicated.

Thank you for your participation.

This survey aims to assess the perceptions and experiences of primary healthcare practitioners in managing common mental health disorders in the Garden Route District. Your honesty in answering the questions is vital to provide accurate insights into authentic experiences. Thank you for your assistance. All surveys are anonymous and facility data is purely for record keeping purposes and will not be included in the research reports.

**Table:**

Date:	________________________________________
Age:	________________________________________
Gender:	○ Male ○ Female ○ Other
Designation/Job Title:	○ Lay Counsellor ○ Registered Counsellor ○ Social worker ○ Psychologist ○ Medical Officer ○ Sessional Doctor ○ Registrar ○ Clinical Nursing Practitioner ○ Psychiatric Nursing Practitioner ○ Professional Nurse ○ Community Health Worker
Subdistrict:	○ Mosselbay ○ George ○ Hessequa ○ Knysna ○ Kannaland ○ Bitou ○ Oudtshoorn
Please select type of facility you are currently employed at.	○ Primary Health Clinic/Satellite Clinic ○ Community Day Centre ○ Hospital Out-Patient Department
Approximately how many patients do you see per day?	________________________________________
Approximately how many of these patients have a mental health disorder?	________________________________________
Have you received training in managing common mental health disorders during your undergraduate program?	○ Yes ○ No ○ Unsure (e.g Depressive disorders, anxiety disorder, alcohol or substance abuse)
If Yes, please indicate most appropriate description of training received	○ Informal Lectures/Group discussions. ○ Rotation in general wards/primary care facilities. ○ Theory and/or formal lectures (module) ○ Clinical rotation in psychiatry. ○ Rotation in dedicated psychiatric ward/facility.
How would you assess your competence at the end of the training to independently recognize, diagnose and manage patients with common mental health disorders?	○ Poor ○ Fair ○ Average ○ Good ○ Excellent (e.g Depressive disorders, anxiety disorder, chronic fatigue, sleep disturbances, unexplained somatic complaints, alcohol or substance abuse)
Have you received any post graduate training in mental health care?	○ Yes ○ No
If yes, please indicate most appropriate description of training received	○ Training seminar/Short course ○ CPD activity/ In-service training ○ Mental health course ○ Module within another course/ speciality ○ Diploma in Mental Health ○ Degree in Mental Health
Please grade your level of interest in the field of mental health care.	○ Completely disinterested ○ Somewhat disinterested ○ Neither interested or disinterested ○ Somewhat interested ○ Very interested
How would you grade the importance of mental health care, in general, within services offered at primary care level? (i.e. Screening, presence of trained practitioners, awareness and signage)	○ Very unimportant ○ Somewhat unimportant ○ Neutral ○ Somewhat important ○ Very important
Which option would describe your emotions the best when you are told that your next patient has a mental health disorder?	○ Anger ○ Frustrated ○ Demotivated ○ Motivated ○ Happy ○ Excited ○ Indifferent ○ Anxious
Please select an option that describes the reason for your emotion the best.	○ Personal reasons regarding mental health disorders (eg. Burn-out, stigma, own mental health status, confidence level, etc.) ○ Environmental/Institutional reasons (workload, prevalence of mental health disorders, etc.) ○ System reason (eg. Referrals, stationary, policies, etc.)
How often do you include mental health screening questions in your consultations?	○ Never ○ Almost never ○ Sometimes ○ Regularly ○ Always
How would you grade your confidence level in being able to recognize a mental health disorder?	○ Complete lack of confidence ○ Low confidence ○ Confident ○ Highly confident ○ Very highly confident
How would you grade your confidence level in making an accurate mental health diagnosis?	○ Complete lack of confidence ○ Low confidence ○ Confident ○ Highly confident ○ Very highly confident
How would you grade your confidence level in managing a patient with a mental health disorder?	○ Complete lack of confidence ○ Low confidence ○ Confident ○ Highly confident ○ Very highly confident
How would you grade your confidence level in appropriately referring a patient with a mental health disorder?	○ Complete lack of confidence ○ Low confidence ○ Confident ○ Highly confident ○ Very highly confident
How would you grade your confidence level in identifying psychotropic medication/s on a patient’s prescription chart?	○ Complete lack of confidence ○ Low confidence ○ Confident ○ Highly confident ○ Very highly confident
Are you aware of the PACK-guidelines for managing common mental health disorders at primary level?	○ Yes ○ No
If yes, please grade your usage of these guidelines.	○ Never ○ Almost never ○ Sometimes ○ Regularly ○ Always
How would you grade your overall experience when referring a patient with a suspected/confirmed mental health disorder to the next level of care?	1- Very Poor 5- Neutral 10- Excellent □□□□□□□□□□□□□□□□□□□□□□□□□□□□□□ (*Place a mark on the scale above*)
Which option best describes your opinion regarding referring a patient with a mental health disorder to the next level of care?	○ It is difficult to reach a referral practitioner ○ It is somewhat challenging to reach a referral practitioner ○ It is easy to reach a referral practitioner (Next level of care: CNP/MO/Mental Health Nurse/Psychiatry MO or Consultant)
Which option best describes your opinion on consultation dates for patients with mental health disorders at the next level of care?	○ Patients WAIT long for consultation dates ○ Patient waiting times are variable ○ Patients DO NOT wait long for consultation dates (Next level of care: MO date at clinic, Mental Health Nurse Clinic, Outreach Psychiatry Clinic, Psychiatry OPD)
Which option will best describe your opinion on receiving feedback regarding referred patients with mental health disorders?	○ I NEVER receive any feedback from the referral practitioner/institution ○ I SOMETIMES receive feedback from the referral practitioner/institution ○ I ALWAYS receive feedback from the referral practitioner/institution
How would you respond to the following statement: A patient with a mental health disorder reaches the appropriate level of care within a reasonable time.	○ Strongly disagree ○ Disagree Neither agree or disagree Agree ○ Strongly agree
How would you respond to the following statement: Mental health awareness in my facility is sufficient.	○ Strongly disagree ○ Disagree ○ Neither agree or disagree Agree Strongly agree Neither agree or disagree
How would you respond to the following statement: My facility and it’s staff display empathy to patients with mental health disorders.	○ Strongly disagree ○ Disagree ○ Neither agree or disagree ○ Agree ○ Strongly agree
How would you respond to the following statement: Managing mental health disorders at primary level care is mostly damage control.	○ Strongly disagree ○ Disagree ○ Neither agree or disagree ○ Agree ○ Strongly agree
How would you respond to the following statement: Mental health services in primary health care do not achieve adequate disease control in patients with mental health disorders.	○ Strongly Disagree ○ Disagree ○ Neither Agree or Disagree ○ Agree ○ Strongly Agree
How would you respond to the following statement: My colleagues have sufficient knowledge about mental health disorders to manage the patients correctly and effectively.	○ Strongly disagree ○ Disagree ○ Neither agree or disagree ○ Agree ○ Strongly agree
How would you respond to the following statement: My workload is too much to offer mental health screening in the consultation.	○ Strongly disagree ○ Disagree ○ Neither agree or disagree ○ Agree ○ Strongly agree
How would you respond to the following statement: The staff at my facility view patients with mental health disorders as frustrating, exhausting and/or troublesome.	○ Strongly disagree ○ Disagree ○ Neither agree or disagree ○ Agree ○ Strongly agree
How would you respond to the following statement: I avoid mental health related complaints within a consultation.	○ Never ○ Almost never ○ Sometimes ○ Regularly ○ Always
How would you respond to the following statement: I would attend training opportunities in managing mental health at primary level care if available	○ Yes ○ No ○ Unsure
